# Zeocin-induced DNA damage response in barley and its dependence on ATR

**DOI:** 10.1038/s41598-024-53264-0

**Published:** 2024-02-07

**Authors:** Jovanka Vladejić, Martin Kovacik, Jana Zwyrtková, Miriam Szurman-Zubrzycka, Jaroslav Doležel, Ales Pecinka

**Affiliations:** 1 Centre of Plant Structural and Functional Genomics, Institute of Experimental Botany of the Czech Academy of Sciences, Olomouc, Czechia; 2 Department of Cell Biology and Genetics, Faculty of Science, Palacký University, Olomouc, Czechia; 3 Institute of Biology, Biotechnology and Environmental Protection, Faculty of Natural Sciences, University of Silesia in Katowice, Katowice, Poland

**Keywords:** Abiotic, Plant genetics, Plant molecular biology

## Abstract

DNA damage response (DDR) is an essential mechanism by which living organisms maintain their genomic stability. In plants, DDR is important also for normal growth and yield. Here, we explored the DDR of a temperate model crop barley (*Hordeum vulgare*) at the phenotypic, physiological, and transcriptomic levels. By a series of in vitro DNA damage assays using the DNA strand break (DNA-SB) inducing agent zeocin, we showed reduced root growth and expansion of the differentiated zone to the root tip. Genome-wide transcriptional profiling of barley wild-type and plants mutated in DDR signaling kinase *ATAXIA TELANGIECTASIA MUTATED AND RAD3-RELATED* (*hvatr.g*) revealed zeocin-dependent, ATR-dependent, and zeocin-dependent/ATR-independent transcriptional responses. Transcriptional changes were scored also using the newly developed catalog of 421 barley DDR genes with the phylogenetically-resolved relationships of barley *SUPRESSOR OF GAMMA 1* (*SOG1*) and *SOG1-LIKE* (*SGL*) genes. Zeocin caused up-regulation of specific DDR factors and down-regulation of cell cycle and histone genes, mostly in an ATR-independent manner. The ATR dependency was obvious for some factors associated with DDR during DNA replication and for many genes without an obvious connection to DDR. This provided molecular insight into the response to DNA-SB induction in the large and complex barley genome.

## Introduction

Cells combat DNA damage caused by various factors such as ultraviolet radiation, pathogens, transposable elements, and replication errors. This is known as the DNA damage response (DDR) and involves a complex network of sensors, transducers, mediators, and effectors^1^. Preservation of the cellular DNA is imperative for the normal progression of the cell cycle and growth. DNA single and double-strand breaks (SSBs and DSBs) are highly toxic forms of damage. SSBs induced by oxidative stress are processed by Poly(ADP-ribose) polymerases (PARPs) and Poly(ADP-ribose) glycohydrolases, while those resulting from TOPOISOMERASE I activity are first processed by Tyrosyl-DNA phosphodiesterase 1 (TDP1). Ligation is carried out by single-strand DNA ligase I, and X-ray repair cross-complementing protein 1 (XRCC1) in plants. DSB repair is more complex, involving canonical non-homologous end joining (cNHEJ) for blunt-ended breaks, and alternative end joining (alt-EJ), single-strand annealing (SSA), or homologous recombination (HR) for staggered ends. Individual pathways are not equal in their repair fidelity. The alt-EJ is error-prone, SSA leads to DNA loss, and only HR enables error-free repair. The alt-EJ pathway is promoted by POLYMERASE Q (TEBICHI), while SSA and HR depend on the damage recognition by the MRE 11, RAD50 and NIJMEGEN BREAKAGE SYNDROME (MRN) complex, which activates ATAXIA TELANGIECTASIA MUTATED (ATM) and ATM AND RAD3-RELATED (ATR) kinases^2^. ATM is activated by DSBs, while ATR has preference for single-stranded DNA. The Arabidopsis *atm* and *atr* single mutants develop normally, but *atm* mutants are partially, and *atm atr* double mutants are fully sterile^3,4^. The *atm* plants are sensitive preferentially to DSB inducers^3^, while *atr* plants are sensitive to agents interfering with replication^4^. Both ATM and ATR phosphorylate key transcription factor SUPPRESSOR OF GAMMA 1 (SOG1) that orchestrates downstream DDR responses in Arabidopsis^5,6^. SOG1 activation leads to transcriptional changes in two-thirds of DDR-responsive genes, including cell cycle regulators and DDR effector proteins^7,8^.

Knowledge on DDR in cereals, including cultivated barley (*Hordeum vulgare* L. subsp. *vulgare*), is limited, and the functional understanding of DDR factors in barley is just beginning. Barley is a genetic model for temperate cereals with a large diploid genome (1C = 5.1 Gbp) and Rabl chromosome organization. Early studies examined DNA repair in barley seeds and embryos using *N*-methyl-*N*-nitrosourea and methyl methane sulfonate treatments^9–12^. Recent research assessed barley DSB repair capacity and pathway choice, revealing the involvement of NHEJ, alt-EJ, and HR pathways and a high (> 81%) coincidence of sister chromatid exchanges indicating a frequent use of sister chromatids as a template for repair^13^. In addition, 148 barley genes related to DNA damage repair response and replication were identified and served as the first dedicated resource for DDR analysis in barley^14^. Also, delayed transcriptomic response to gamma-radiation was analyzed using microarrays^15^. In this study, dry seeds were irradiated, then imbibed for 2, 24 or 48 h and subsequently the expression changes were analyzed. The low irradiation dose affected expression of phytohormones, late embryogenesis abundant proteins and cell wall components, while the high dose caused expression changes indicating cell cycle arrest, activation of DNA damage repair, and antioxidants. A barley ATR mutant (*hvatr.g*) was identified and characterized in the HorTILLUS tilling population^16^. The *hvatr.g* plants resemble wild-type (WT) but exhibit increased DNA damage levels even under normal conditions. They also have a deregulated cell cycle and continue dividing in the presence of toxic concentrations of aluminum^16^. The *hvatr.g* plants show reduced efficiency of DNA repair during DNA-replication stress, confirming the involvement of the barley ATR homolog in DNA damage repair^17^.

Here, we describe the effects of radiomimetic agent zeocin on the growth of barley WT and *hvatr.g* mutant plants. We devised a methodology that allows robust testing of barley sensitivity to genotoxic stress. Subsequently, we performed genome-wide transcriptomic analysis after DNA damage induction in WT and *hvatr.g* plants and identified sets of genes that are misregulated by zeocin treatments in ATR dependent or independent manner. Our analyses were supported by the newly developed list of 421 barley DDR genes.

## Results

### Establishing barley in vitro DNA damage treatment

We developed a protocol to assess barley plant sensitivity to DNA damaging treatments. Approximately 20 dissected mature barley embryos were cultured on 100 ml of regular ½ MS medium in plastic cultivation boxes, resulting in synchronized germination approximately 24 h after initiation of cultivation.

**Figure 1:**
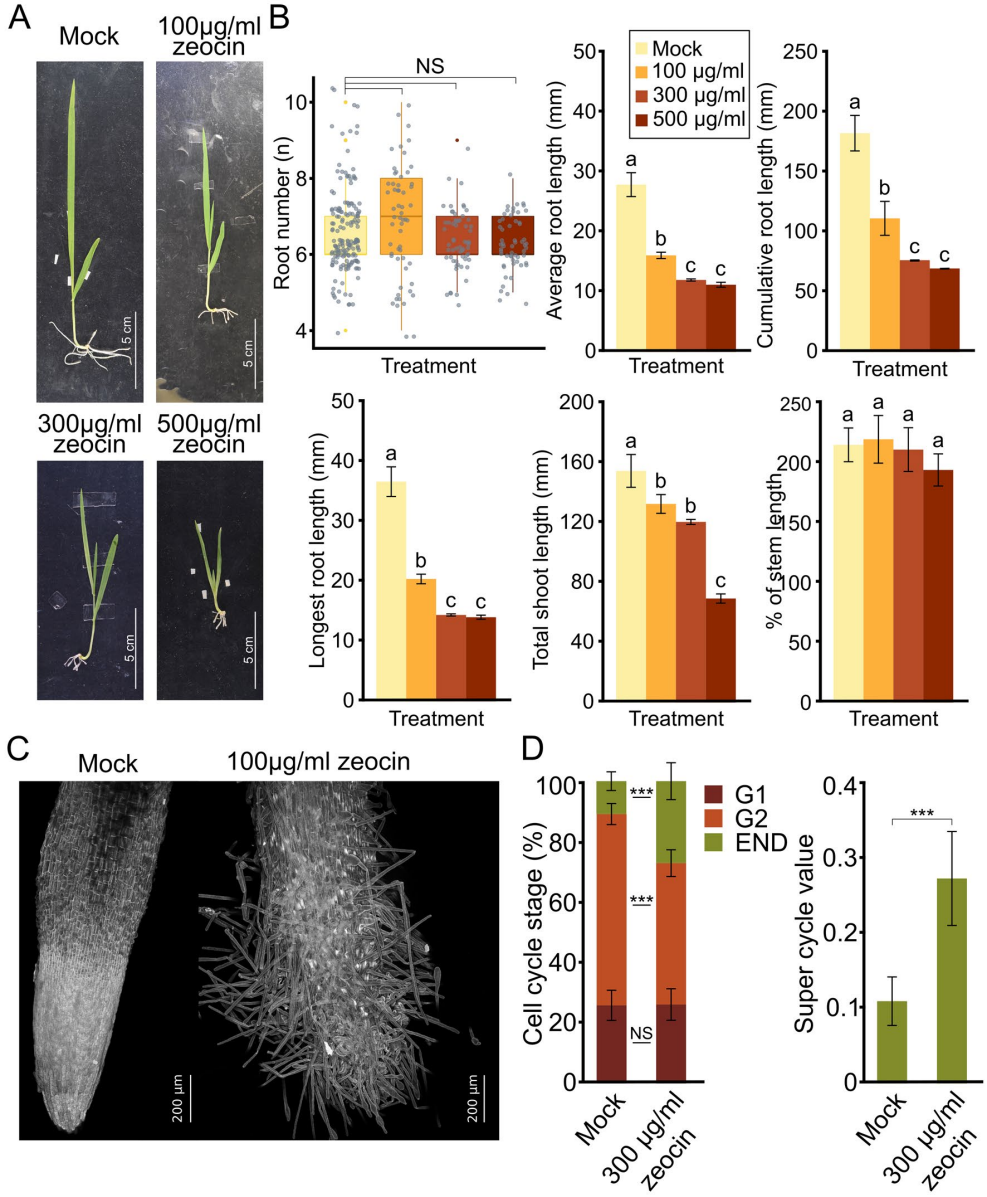
Wild-type barley (*cv.* Golden Promise) parameters in response to zeocin treatment. (**A**) Representative phenotypes of barley seedlings grown for 7 days on ½ MS (mock) and zeocin concentrations (100, 300, 500 μg/ml) containing media. Scale bars = 5 cm. (**B**) Effect of zeocin treatments on barley seedling growth. Error bars indicate the standard deviation between the means of three biological replicates. The letters above columns indicate similarities between samples. The same letters indicate samples that were not significantly different in one-way ANOVA with post hoc Tukey’s test (*P* < 0.05). Kruskall-Wallis H-test with post hoc Conover-Iman test of multiple comparisons with Benjamini–Hochberg procedure (*P* < ½ α, α = 0.05) were used to analyze differences in root numbers. NS, not significant. (**C**) Representative confocal microscopy maximal projection images of the root surface in plants grown in mock conditions and plants treated with 100 μg/ml zeocin. Scale bar = 200 μm. (**D**) Percentage of nuclei at specific cell cycle stages found in the root tips of plants grown on mock and medium with 300 μg/ml zeocin. Super cycle value describing the amount of nuclei in endoreduplication (END). Statistical relevance distinguished by two-sample T-test, (*P* < 0.05). *P < 0.05, ***P < 0.001.

We established an effective DNA damage treatment for WT barley cultivar (*cv*.) Golden Promise) using zeocin dilution series (100, 300, and 500 μg/ml). Zeocin is an antibiotic that causes DNA single and double strand breaks in a ratio 9:1, respectively, in experiments using Phage DNA^18^. Golden Promise strain embryos (n = 18–20 per experimental point) were cultured on mock and zeocin-containing media for 14 days (Fig. 1A). Phenotypic parameters were analyzed to identify informative traits. No significant differences were observed in root number between mock and zeocin treatments (Fig. 1B). However, the average root length showed a significant reduction, from 27 ± 2 mm (mock) to 16 ± 0.6, 11.9 ± 0.3, and 11.1 ± 0.5 mm (42%, 60%, and 60% reduction) with increasing zeocin concentrations (Fig. 1B). Similar trends were observed for the longest root length and cumulative root length parameters (Fig. 1B). The longest root length decreased from 36 ± 2.5 mm (mock) to 20 ± 0.9, 14 ± 0.3, and 14 ± 0.4 mm in individual zeocin treatments (Fig. 1B). Cumulative root length decreased significantly from 181 ± 15.2 mm (mock) to 110.5 ± 14.5, 76 ± 1, and 69.1 ± 0.5 mm with increasing zeocin concentrations. Additionally, two shoot parameters were assessed (Fig. 1B). The total shoot length (from the base of hypocotyl to the tip of the longest leaf) decreased from 153.2 ± 11.2 mm (mock) to 131.1 ± 6.6, 119.5 ± 2.0, and 68.8 ± 3.4 mm after zeocin treatments, corresponding to 14.2%, 22%, and 55.1% reduction, respectively (Fig. 1B). The length of hypocotyl relative to the whole stem length did not show significant differences between treatments (Fig. 1B).

Based on reduced root growth, we examined root apices and observed elongated root hairs and the absence of meristematic zone and the typical conically shaped root tip in 100 μg/ml zeocin-treated plants (n = 3) (Fig. 1C). This suggests premature differentiation of barley root apical meristem tissues upon DNA damage. DNA damage response often affects cell cycle dynamics^19^. Flow-cytometric DNA content measurements in 7-day-old mock-treated plants (n = 20) showed approximately 25.8 ± 5.2% 2C (G0/G1), 63.3 ± 3.7% 4C (G2), and 10.9 ± 3.3% 8C (endoreduplicated) nuclei in root apices (Fig. 1D). After 300 μg/ml zeocin treatment (n = 20), there was a 27% reduction in G2 nuclei (46.7 ± 4.6%), and endoreduplicated nuclei (8C) were 2.5 times more frequent (27.1 ± 6.3%). The super cycle value^20^, indicating the percentage of endoreduplicated nuclei, showed a significant 2.5-fold increase after zeocin treatment (Fig. 1D).

Collectively, we observed reduced root growth and shorter stems after zeocin treatment, with 100 μg/ml zeocin being sufficient for significant changes. Thus, we successfully established zeocin-induced in vitro DNA damage conditions in barley.

### *Hvatr.g* plants are sensitive to zeocin treatment

**Figure 2:**
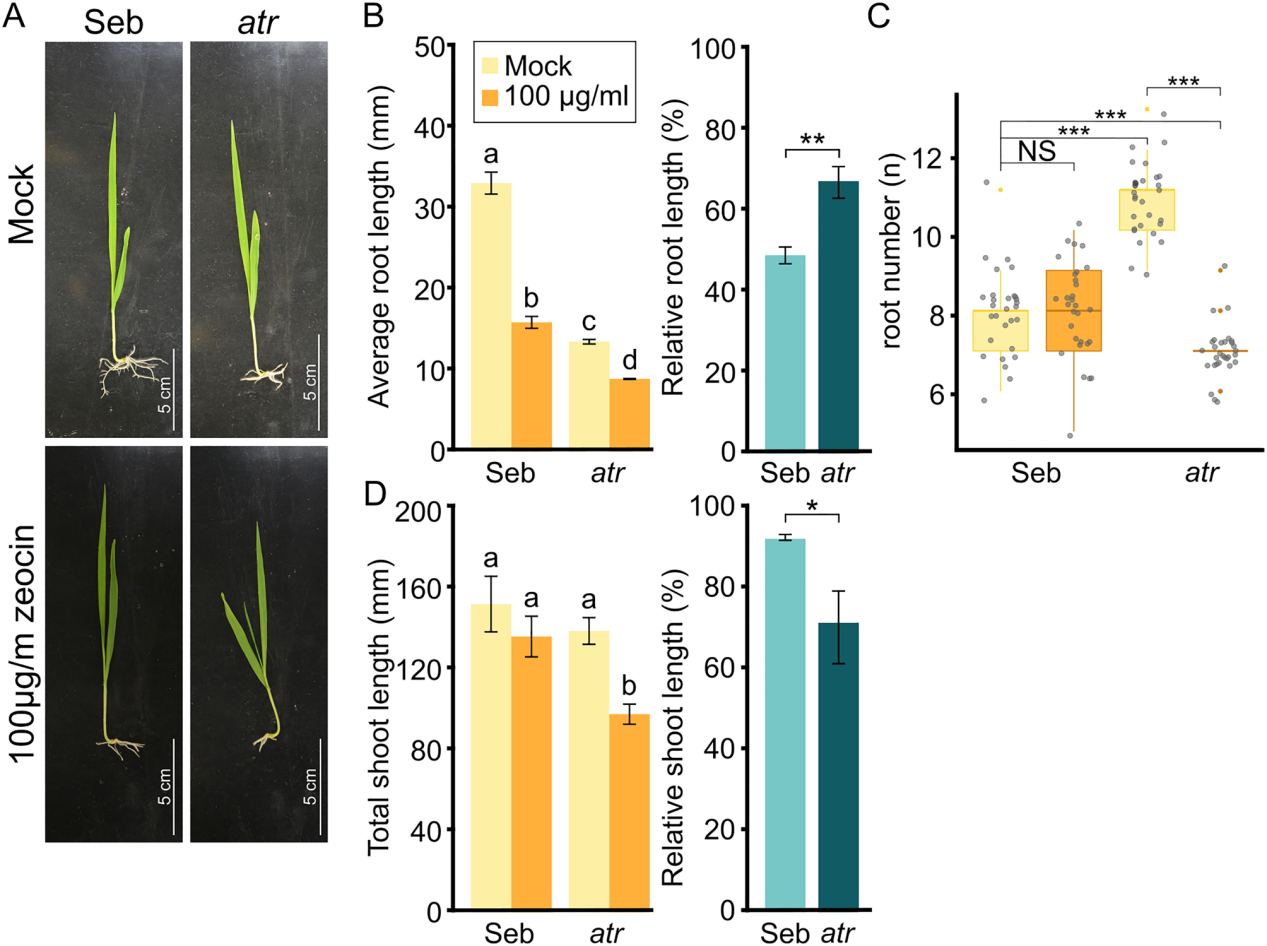
Barley *atr* mutant plants are sensitive to zeocin treatment. (**A**) Representative images of wild-type cultivar Sebastian (Seb) and *atr* mutant plants treated with 100 μg/ml zeocin, or untreated (mock). Scale bars = 5 cm. (**B**) Effect of zeocin treatment on Seb and *atr* plants’ root growth. Error bars indicate the standard deviation between the means of three biological replicates. For the absolute values represented the letters above columns indicate similarities between samples. The same letters indicate samples that were not significantly different in one-way ANOVA with post hoc Tukey’s test (*P* < 0.05). For the assessment of the statistical significance of the relative values the two-sample T-test was used, *P < 0.05, **P < 0.01. (**C**) Number of roots in wild-type (Seb) and *atr* plants grown in mock and genotoxic conditions. Kruskall–Wallis H-test with post hoc Conover-Iman test of multiple comparisons with Benjamini–Hochberg procedure (*P* < ½ α, α = 0.05) was used to analyze differences in root numbers. NS, not significant, ***P < 0.001. (**D**) Effect of zeocin treatment on Seb and *atr* plants’ shoot growth. Error bars indicate the standard deviation between the means of three biological replicates. For the absolute values represented the letters above columns indicate similarities between samples. The same letters indicate samples that were not significantly different in one-way ANOVA with post hoc Tukey’s test (*P* < 0.05). For the assessment of the statistical significance of the relative values the two-sample T-test was used, *P < 0.05.

To explore barley’s DNA damage response (DDR) in the context of DNA damage signaling, we utilized a loss-of-function mutant allele of ATR kinase (*hvatr.g*) in the Sebastian background^21^. Previous study on hydroponically grown *hvatr.g* plants demonstrated shorter roots compared to *cv.* Sebastian (WT)^17,21^. We observed a similar trend in plants grown from rescued embryos (n = 9–10) on solid media in vitro (Fig. 2A). Cultivar Sebastian was used as wild-type, and its phenotypic responses were comparable to those of *cv.* Golden Promise. Under mock conditions, *hvatr.g* plants exhibited a 60% reduction in root length compared to WT plants (13.4 ± 0.3 mm and 32.8 ± 1.4 mm, respectively) (Fig. 2B). Following 100 μg/ml zeocin treatment, the average root length was 15.8 ± 0.8 mm for WT plants and 8.9 ± 0.1 mm for *hvatr.g* plants (Fig. 2B). This corresponded to a 51.5% reduction for zeocin-treated WT plants and a 33.8% reduction for zeocin-treated *hvatr.g* plants (Fig. 2B). The greater reduction in WT plants likely indicates their higher potential for root shortening compared to *hvatr.g* plants, which already had significantly reduced root length. Similar results were obtained for cumulative root length and the longest root (Supplemental Fig. 1). The median root number was significantly higher in *hvatr.g* plants (median root number = 11) compared to WT plants (median root number = 8) (Fig. 2C). After zeocin treatment, WT plants maintained the median number of roots (median root number = 8), while the number decreased (median root number = 7; 36% reduction) in *hvatr.g* plants. Additionally, we analyzed plant height (Fig. 2D). Zeocin-treated WT plants exhibited a non-significant reduction of 10.4% (*P* > 0.05) in height (135.0 ± 10.3 mm) compared to mock-treated plants (150.8 ± 14 mm), whereas zeocin-treated *hvatr.g* plants experienced a 29.4% reduction (97.1 ± 5.3 mm) compared to mock-treated *hvatr.g* plants (137.6 ± 6.9 mm).

These findings suggest that total shoot length is the most informative characteristic for assessing the DNA damaging effects of zeocin on barley *hvatr.g* plants.

### Identification of barley homologs of DDR genes

To prepare for the transcriptomic analysis, we identified barley homologs of known DNA damage response (DDR) genes. Using the published list of 321 Arabidopsis DDR genes^22^, we performed a series of BLASTs and selection steps, resulting in the discovery of 421 barley homologs (Supplemental Dataset 1). Visual inspection revealed the absence of *BRCA1* due to a lower homology. This gene was added manually and made the final set of 421 barley homologs. Approximately 50% of the identified proteins (n = 204) were coded by single-copy homologs, such as *ATM*, *ATR*, *WEE1*, *BRCA1*, *BARD*, and *RAD51*. We also observed an expansion of *PCNA2*ed family to 11 members in barley. Arabidopsis *PCNA2* interacts with the translesion synthesis polymerase POLH and is involved in the repair of UV-induced damage^23^.

**Figure 3:**
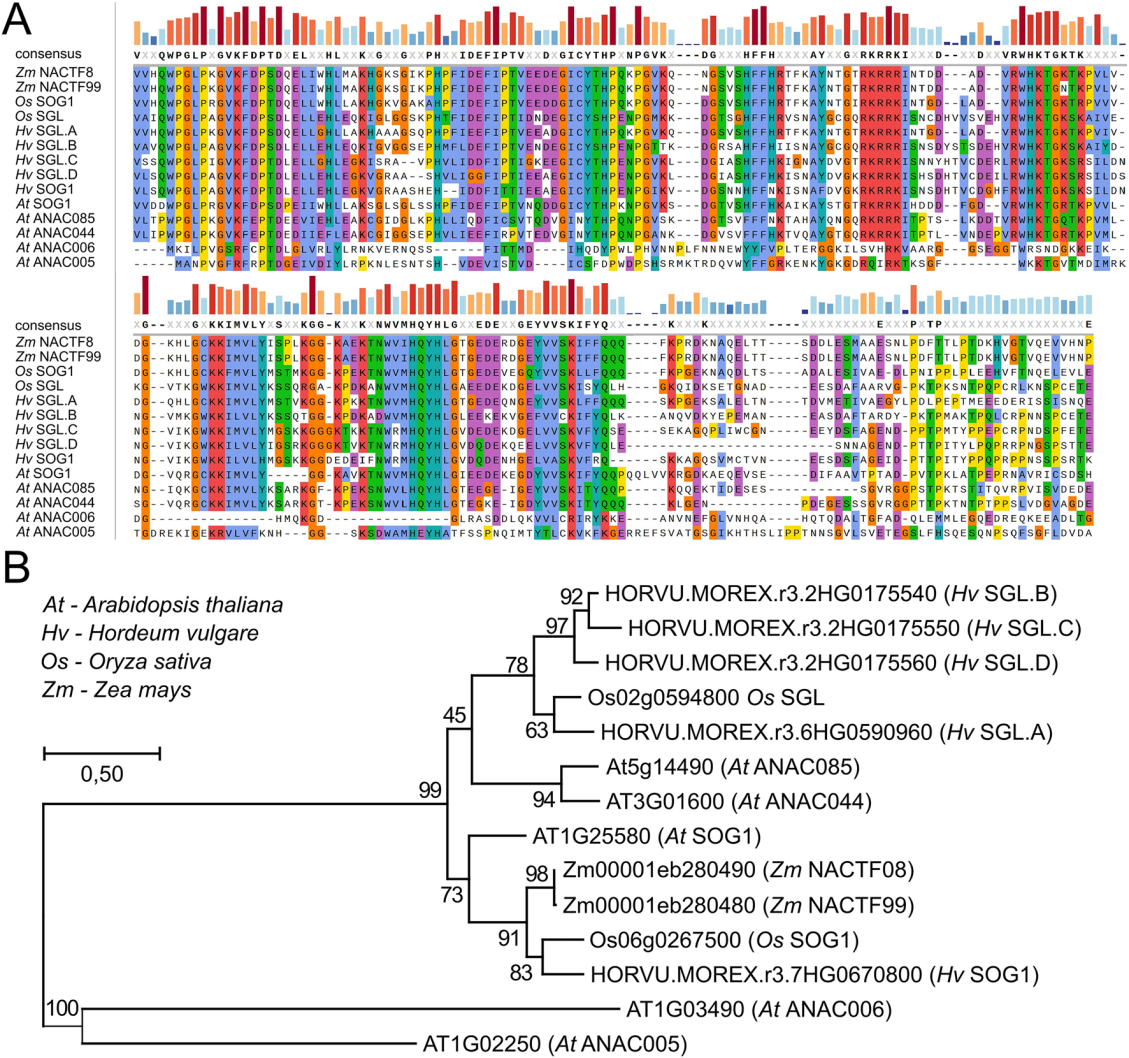
Phylogenetic analysis of barley SOG1 and SGLs. (**A**) Multiple sequence alignment of NAC protein domains present in five barley SOG1 protein candidates with the NAC domains of *Arabidopsis thaliana* (*At*) SOG1, ANAC044, and ANAC085, *Oryza sativa* (*Os*) SOG1 and SOG1-like (SGL), and *Zea mays* (*Zm*) NACTF8 and NACTF99 proteins by MUSCLE. Amino acids are highlighted based on consensus sequence and their physicochemical properties. (**B**) Phylogenetic Maximum Likelihood tree built based of the multiple sequence alignment, with *Arabiodopsis* ANAC005 and ANAC006 NAC protein domains used as an outgroup. Bootstrap values are shown next to the branches, distance scale = 0.5.

Of special interest was the master regulator of plant DDR SOG1 and its potential homologs. SOG1 is a member of a large NAC (NAM, ATAF1/2, and CUC2) family of transcription factors in plants and a list of 167 barley NAC domain containing proteins was published, highlighting HORVU.MOREX.r3.7HG0670800 (alias HORVU7Hr1G042420) as the most likely homolog of Arabidopsis ANAC008 (*At*SOG1)^24^. On contrary, another gene HORVU.MOREX.r3.6HG0590960 (alias HORVU6Hr1G053540) was suggested as *HvSOG1* based on upregulation upon DNA damage treatment^25^. Therefore, we analyzed SOG1 in barley in more detail. Via BLAST we found five barley genes that shared a significant homology with the *AtSOG1* (Supplemental Dataset 1). Three were analyzed previously^24^. To assess their relationships, we performed a phylogenetic study using five SOG1 and SOG1-LIKE (SGL) factors from barley, two from rice, two from maize and three from Arabidopsis (Fig. 3). The Arabidopsis factors included *At*SOG1/*AtANAC8* and two closely related transcription factors *At*ANAC44 and *At*ANAC85 that were also shown to play a role in DNA damage response^26^.

Multiple sequence alignment and phylogenetic tree revealed three main clades, (i) the *At*ANAC005 and *At*ANAC006 outgroup, (ii) the SOG1 and (iii) the SGL clades. The SOG1 clade contained Arabidopsis SOG1, rice *Os*SOG1, both maize SOG1 candidates (*Zm*NACTF99 and *Zm*NACTF08) and HORVU.MOREX.r3.7HG0670800, suggesting it as the most likely barley SOG1 ortholog (Fig. 3). The SGL clade, defined based on a lower 45% confidence at its root but high confidence at the branches, contained *At*ANAC44 and *At*ANAC85, rice *Os*SGL, and the four remaining barley candidates. The barley protein HORVU.MOREX.r3.6HG0590960 was most closely related (63%) to the *Os*SGL; therefore, we named it as *Hv*SGL.A. The remaining three candidates HORVU.MOREX.r3.2HG0175540 (SGL.B), HORVU.MOREX.r3.2HG0175550 (SGL.C), and HORVU.MOREX.r3.2HG0175560 (SGL.D) showed 58 to 66% protein identity and their consequent gene identifiers suggest that they are inparalogs that arose by a local tandem triplication.

Hence, we developed a list of 421 barley homologs of known DDR genes and found a single *SOG1* ortholog and four *SGL* genes (*SGL.A*, *SGL.B*, *SGL.C* and *SGL.D*) in barley.

### Transcriptomic responses of WT barley to zeocin treatments

**Figure 4:**
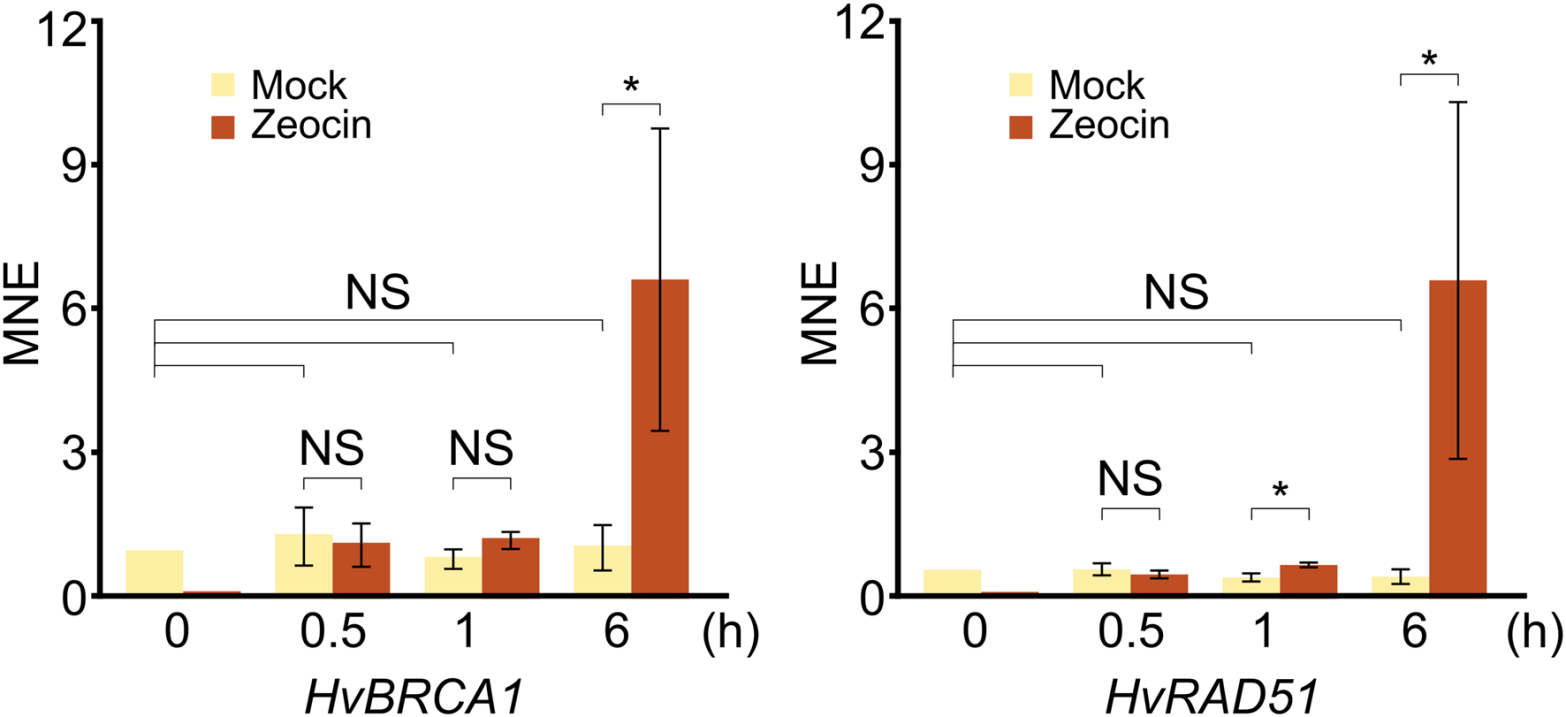
Barley *BRCA1* and *RAD51* are transcriptionally upregulated after zeocin treatment in Golden Promise wild-type. Reverse transcription-quantitative PCR values were normalized to the expression of *UBC2* as mean normalized expression (MNE). Error bars indicate standard deviation of three biological replicates. Statistical significance of the difference presented was tested by two-sample T-test, (*P* < 0.05). *P < 0.05.

We analyzed the transcriptional response of barley plants to zeocin treatments. Initially, we examined the expression of barley *BRCA1* and *RAD51* genes in seven-day-old WT (*cv.* Golden Promise) plants treated with 300 μg/ml zeocin for 0, 0.5, 1, and 6 h using RT-qPCR. Both *BRCA1* and *RAD51* showed significant up-regulation, with BRCA1 exhibiting a six-fold increase and RAD51 showing an almost 11-fold up-regulation at the 6-h time point (Fig. 4). Subsequently, we conducted RNA-seq analysis of root apices isolated from *cv.* Sebastian WT and *hvatr.g* plants treated with 0 (mock) and 500 μg/ml zeocin for 6 h, aiming to identify differentially expressed genes (DEGs).

**Figure 5:**
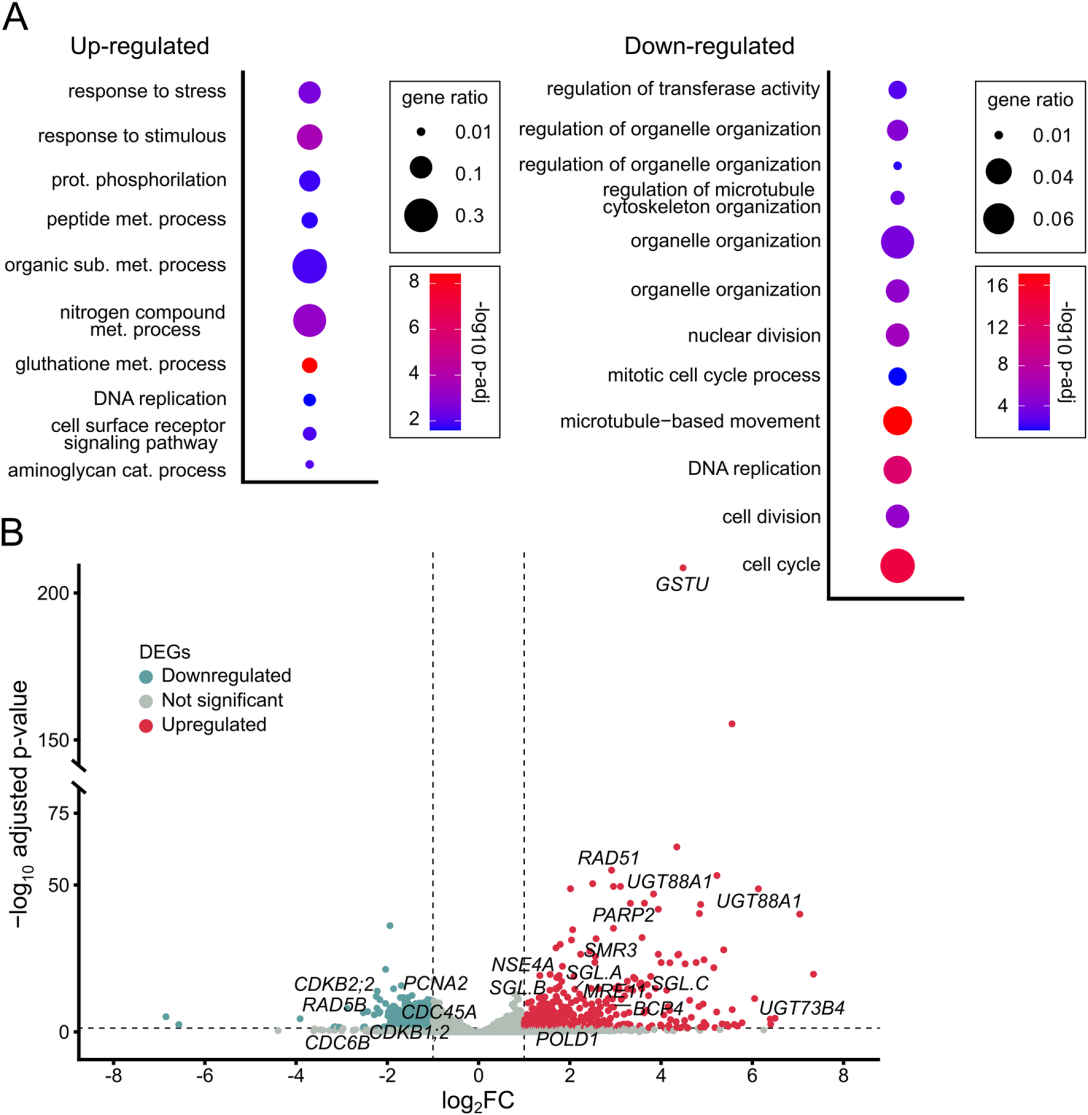
Zeocin effect on gene expression in wild-type barley. (**A**) Biological processes enriched among significantly up-regulated and down-regulated genes in zeocin versus mock treated wild-type (Sebastian) plants. Redundant GO terms were removed manually, based on *P*-value. The full list of GO terms can be found in Suppl. Dataset 3. Statistical significance was determined by Fisher’s one-tailed test with g:SCS algorithm correction. Gene ratio represents the number of genes found in the category compared to the total number of genes in the query. (**B**) Volcano plot representing all genes detected as differentially expressed in wild-type (Sebastian) plants treated with zeocin relative to mock treatment. Horizontal dashed line indicates genes passing *P* < 0.05. Vertical dashed lines separate genes with log_2_ Fold Change ≤ − 1 (blue) or log_2_ Fold Change ≥ 1 (red). Specific marker genes were highlighted by names.

In zeocin-treated versus mock-treated WT plants, we identified 719 DEGs, including 404 significantly up-regulated genes and 315 down-regulated genes (Supplemental Dataset 2). Gene ontology analysis of the up-regulated DEGs revealed their involvement in biological processes such as glutathione metabolic process (GO:0006749), response to stimulus (GO:0050896), response to stress (GO:0006950), and defense response (GO:0006952) (Fig. 5A; Supplemental Dataset 3). Notably, many up-regulated DEGs were associated with oxidative stress signaling (Fig. 5B), including 14 *GLUTATHIONE S-TRANSFERASEs* (*GSTU*) and 14 *UDP*-*GLUCOSYLTRANSFERASEs* (*UGT*) involved in reactive oxygen species detoxification. Other oxidative stress-related genes, such as *OXIDATIVE STRESS 3*, reductases, and a homolog of *CATION EXCHANGER 5*, were also identified. The observed oxidative stress response can be attributed to the radiomimetic activity of zeocin, which generates hydrogen peroxide upon interaction with a metal ion, leading to oxidative damage to DNA and other cellular components^27^.

We found 7.6 and 2.8-fold up-regulation for *RAD51* and *BRCA1* in zeocin-treated WT plants, respectively, indicating activation of DDR also in our RNA-seq experiment. Subsequently, we inspected the full lists of significantly down-regulated and up-regulated DDR candidates. In accordance with the phenotypic data, zeocin treatment had a profound inhibitory effect on the cell cycle, including DNA replication and mitotic cell division. We found significant down-regulation for six *CYCLINs* (*CYC*) (*CYCB1;2.A-C*, *CYCB1;3.A-B*; *CYCB2;3.B*) and two *CYCLIN DEPENDENT KINANSES* (*CDKs*) *CDKB1;2* and *CDKB2;2*, whose Arabidopsis homologs promote G1/S and G2/M transitions, respectively.

Although the GO term analysis indicated genes related to DNA replication are both up- and down-regulated, a more detailed analysis revealed repressive effects of zeocin treatment on DNA replication. Seven genes marked by the GO term analysis as involved in DNA replication were up-regulated by zeocin treatment: *RPA1*, *RPA2*, *RAD51*, *DNA POLYMERASE ZETA SUBUNIT REV3*, and *RIBONUCLEOSIDE-DIPHOSPHATE REDUCTASE*. We argue that the up-regulation of these genes should not be taken as indication for active replication but rather as a sign of its repression and ongoing DDR. The RPA subunits play roles in both DNA replication and repair, DNA POLYMERASE ZETA and RAD51 are primarily associated with DDR, and RIBONUCLEOSIDE-DIPHOSPHATE REDUCTASE is involved in nucleotide synthesis. Next, we found down-regulation of almost all genes coding subunits of the Minichromosome maintenance complex (MCM), DNA primase and REPLICATION PROTEIN A complex. The reduced replication was recognizable also by looking at the expression of histones and replication-coupled chromatin modifiers. In total 63 *HISTONE* genes were down-regulated, including 15 copies of *H2A* in several variants, 9 copies of *H2B*, 23 copies of *H3.1* and 16 copies of *H4*. The downregulated *H2A* variants included one copy of *H2A.Z* and two copies of DDR-associated variant *H2A.X*. This is in line with down-regulation of CAF-1 histone chaperone subunit coding gene *FAS1*. Zeocin-induced down-regulation of replication coupled chromatin modifiers included *VARIANT IN METHYLATION 1* (*VIM1*), and two histone methyltransferases (one uncharacterized and the other homologous to *ARABIDOPSIS TRITHORAX-RELATED PROTEIN 6*). There were also several DDR-associated down-regulated genes, most notably barley homolog of *BARD1*.

Zeocin up-regulated genes pointed to specific directions. The up-regulation of two *PARP* genes and *MRE11* homologs indicated presence of both SSBs and DSBs, respectively. We also saw up-regulation of three RPAs: *RPA70C.C*, *RPA2.K* and *RPA1A* that suggest an increased amount of single stranded DNA. At the level of DDR signaling, we found up-regulation of three *SGL* factors *SGL.A*, *SGL.D*, and *SGL.C*, but not *SOG1*. The up-regulation of barley homolog of Arabidopsis CDK inhibitor *SMR3* is in agreement with the alterations in cell cycle and enhanced levels of endoreduplication. The other responses, presumably downstream of SOG1/SGLs involved several genes known to code factors involved in the HR: *RAD51*, *BRCA1*, *RecQL3* helicase, the SMC5/6 complex subunit *NSE4.A*, SMC hinge domain protein *GAMMA-IRRADIATION AND MITOMYCIN C INDUCED 1* (*GMI1*), or *BRCT5 DOMAIN CONTAINING PROTEIN 1* (*BCP1*).

The comparison of gene expression in zeocin-treated and untreated WT plants showed activation of oxidative stress response, DDR, signatures of halted replication and shift towards endoreduplication cycle.

### Effects of *hvatr.g* on gene expression under mock conditions

**Figure 6:**
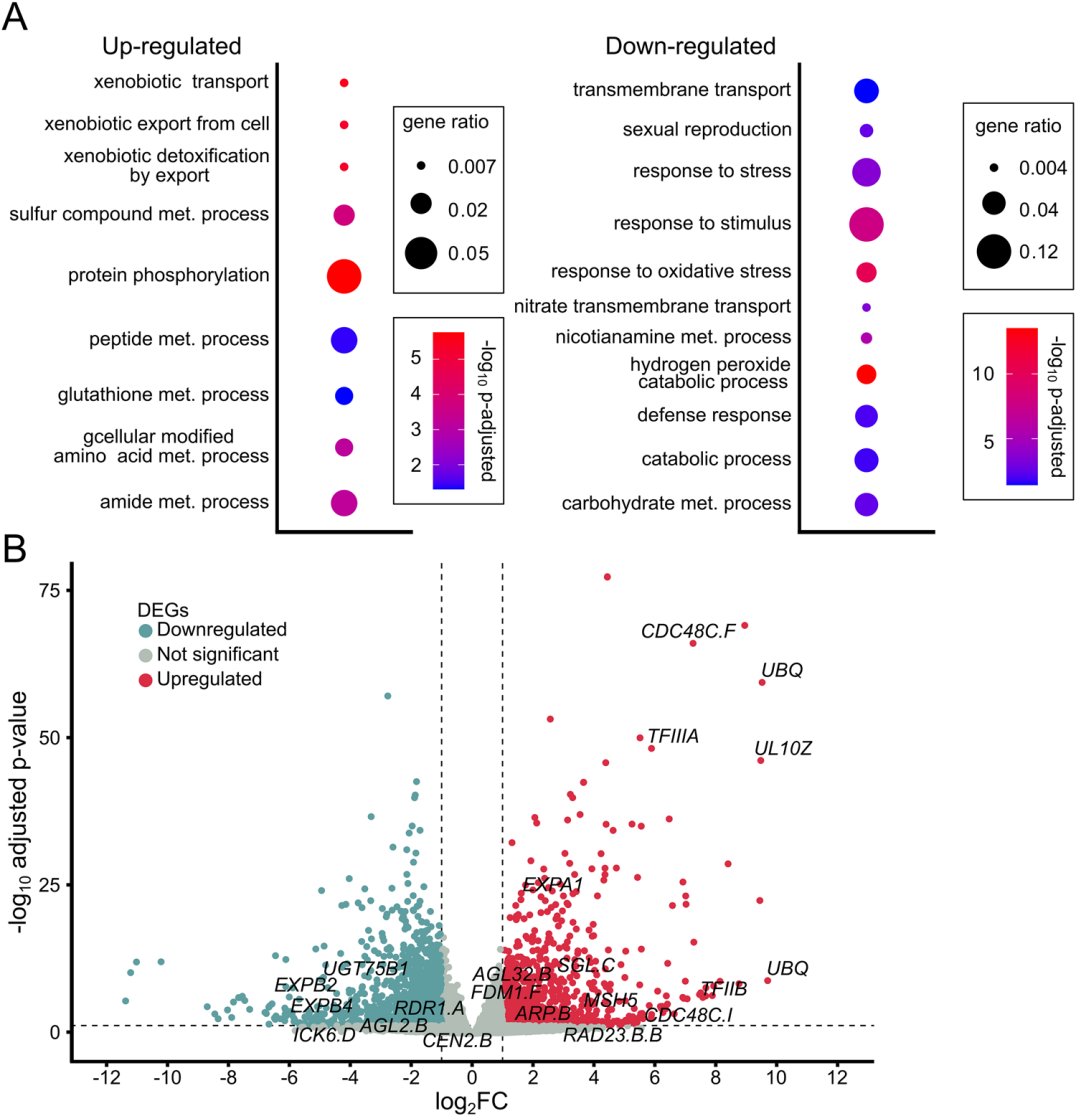
Effects of *ATR* mutation on gene expression in barley. (**A**) Gene enrichment analysis for biological processes in *atr* mutant compared to wild-type (Sebastian). Redundant GO terms were removed manually, based on *P*-value. The full list of GO terms can be found in Suppl. Dataset 3. Statistical significance was determined by Fisher’s one-tailed test with g:SCS algorithm correction. Gene ratio represents the number of genes found in the category compared to the total number of genes in the query. (**B**) Volcano plot representing all genes detected as differentially expressed in *atr* plants relative wild-type (Sebastian) plants. Horizontal dashed line indicates genes passing *P* < 0.05. Vertical dashed lines separate genes with log_2_ Fold Change ≤ − 1 (blue) or log_2_ Fold Change ≥ 1 (red). Specific marker genes were highlighted by names.

We examined the effects of *ATR* mutation on transcriptome by comparing expression in *hvatr.g* (Fig. 6). The *hvatr.g* plants showed a significant (adjusted-*p* ≤ 0.05 and a log_2_ fold change ≤ − 1 or log_2_FC ≥ 1) up-regulation of 1150 and down-regulation of 1457 genes relative to WT plants (Supplemental Dataset 2). Gene ontology analyses of the up-regulated genes suggested a connection to only a few categories of biological processes, most related to protein production and/or modifications, as in GO:0006749 (glutathione metabolic process), GO:0006468 (protein phosphorylation), or GO:0006575 (sulfur compound metabolic process). Additional gene categories include xenobiotic export and transport from cell (GO:0046618, GO:0042908). Categories of down-regulated processes show a miss-regulation of transmembrane transport, especially concerning nitrate (GO:0015706, GO:1902025) (Fig. 6A, Supplemental Dataset 3).

Among the most significantly upregulated transcripts was 64-fold upregulated *TFIIIA* (Fig. 6B), which is known to regulate *5S rDNA* transcription. Furthermore, *hvatr.g* plants had over 182-fold higher expression of *TFIIB* that is involved in the formation of the RNA polymerase II (POL II) transcription preinitiation complex. A very prominent group of the top up-regulated genes were related to ubiquitinylation: *UBIQUITINs*, *UBIQUITIN EXTENSION PROTEINS* and *F-BOX PROTEINS*. The *hvatr.g* plants showed also up-regulation in genes of the DDR pathway like *SGL.C*, suggesting increased genome instability even at mock conditions. Furthermore, there was an up-regulation of the *MutS* homolog 5 (*MSH5*) that was implicated in mismatch repair in Arabidopsis^28^ and nucleotide excision repair and proteolysis associated factors *RADIATION SENSITIVE 23* (*RAD23*) and *CELL DIVISION CYCLE* (*CDC48*).

The strongly down-regulated genes in *hvatr.g* were eight copies of *NICOTIANAMINE SYNTHASE* (Supplemental Dataset 2). These genes should be strongly expressed in roots, where it regulates intake of iron^29^. In rice it was shown to be under direct control of NAC-family proteins during drought stress^30^. The same pathway in rice up-regulates the expression of genes involved in membrane modification genes and transport^30^. Even more remarkable was the decrease in the expression of transmembrane transporters. Among the 71 down-regulated transmembrane transport-related genes, 25 are associated with the transport of nitrogen-based compounds (Supplemental Fig. 3B). Comparison of up-regulated and down-regulated genes with products involved in nitrogen transport or metabolism, confirmed this further (example genes Supplemental Fig. 3A, full gene list Supplemental Dataset 4). The most prevalent down-regulated genes in this group were *HIGH AFFINITY NITRATE TRANSPORTERS* and *NRT1/PTR FAMILY TRANSPORTERS* (6 genes each).

In summary, *hvatr.g* plants show a well notable pattern of transcriptional changes that includes a mix of DDR, transcriptional and translational responses. The groups of up- and down-regulated genes also indicate enhanced turnover of genic products from the transcription, through protein synthesis, modification and degradation.

### Differences in reaction to zeocin caused by *hvatr.g* mutation

**Figure 7:**
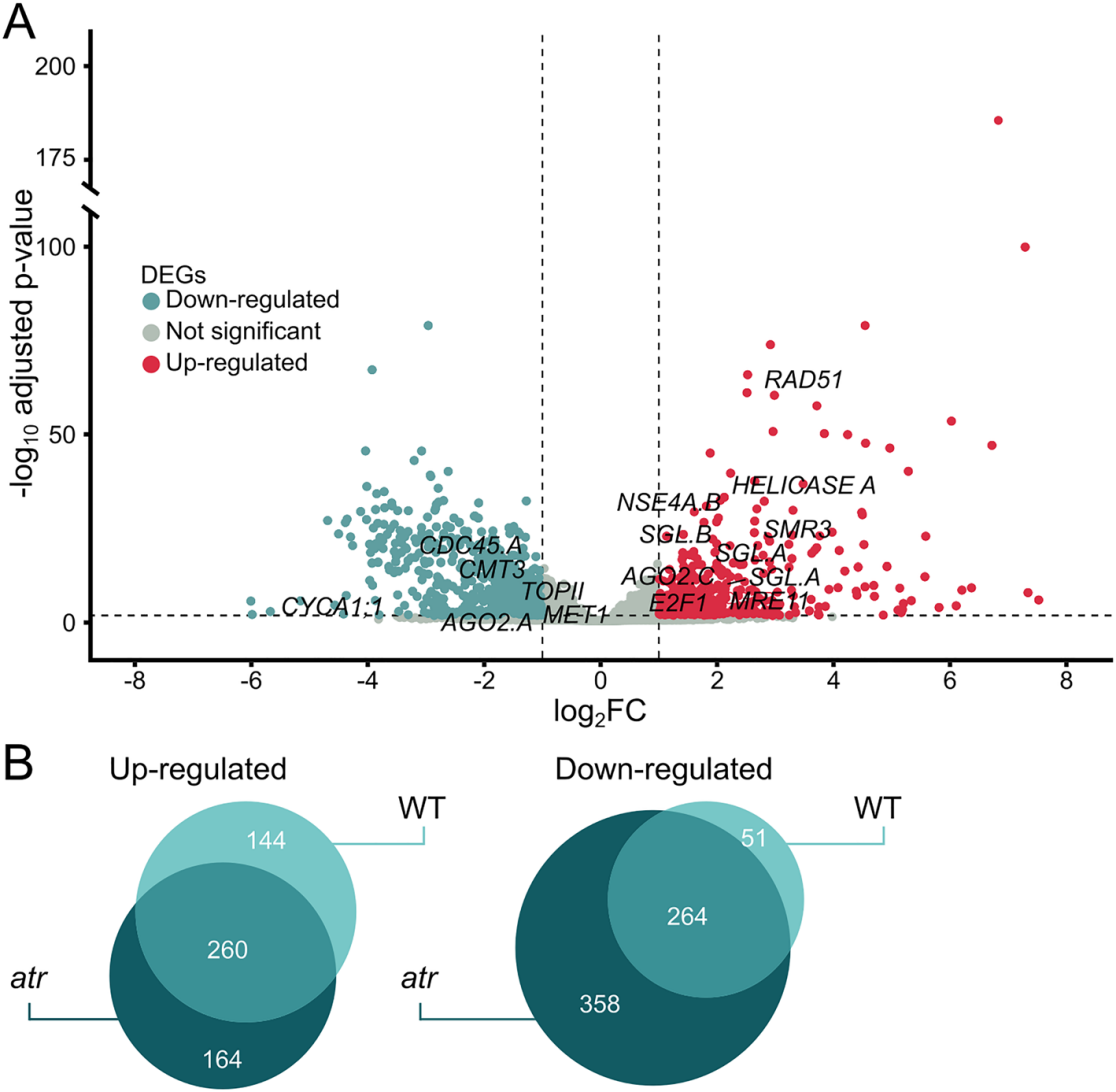
Response of *atr* mutant plants to zeocin treatment. (**A**) Volcano plot representing all genes differentially expressed in zeocin-treated versus mock-treated *atr* mutant plants. Horizontal dashed line indicates genes passing *P* < 0.05. Vertical dashed lines separate genes with log_2_ Fold Change ≤ − 1 (blue) or log_2_ Fold Change ≥ 1 (red). Specific marker genes were highlighted by names. (**B**) Venn diagrams showing differentially expressed genes after zeocin treatment in wild-type and *atr* mutant plants relative to the mock treatment of the same genotype.

To assess the DNA damage response in plants lacking the functioning ATR kinase, we looked on differentially expressed genes in zeocin and mock-treated *hvatr.g* plants. There were in total 424 genes up-regulated and 622 genes down-regulated in zeocin-treated compared to mock-treated *hvatr.g* plants (Fig. 7A, Supplemental Dataset 2, Supplemental Fig. 2). Gene enrichment analysis showed categories similar to those described in zeocin-treated WT plants (Supplemental Fig. 3). To filter for the ATR-specific responses, we visualized the data as Venn diagrams of genes with significantly changed expression by zeocin-treatment in WT and *hvatr.g* plants (Fig. 7B, Supplemental Dataset 5).

The majority of zeocin-induced transcriptional changes in WT were ATR-independent with 260 genes up-regulated (64.4% out of 404) and 264 genes down-regulated (83.8% out of 315) (Fig. 7B, WT and overlap). This included all major up-regulated DDR genes related to SSB repair (*PARP*), translesion synthesis (*REV3*), direct hydrolysis (*TDP*) and HR (*MRE11, SGL.D, SGL.C, SMR3, RAD54, BRCA1, BCP1, BCP4, GMI1, NSE4.A*). Similarly, genes down-regulated by zeocin treatment in ATR-independent manner included many positive regulators of cell cycle, replication factors and histones (Supplementary Dataset 4). When looking on dependence of transcriptional change on ATR upon zeocin treatment, we found 144 up-regulated genes (35.6% out of 404) and 51 down-regulated genes (16.2% out of 315). The DDR associated genes up-regulated in ATR dependent manner included *RECQ HELICASE 3 (RECQL3)* and *DNA POLYMERASE DELTA catalytic SUBUNIT 1* (*POLD1*). Both proteins are involved in processing lagging strand during replication where RECQL3 unwinds it and shows a substrate preference for nicked Holliday junctions^31^ and POLD performs its synthesis^32^.

Finally, the last category represented zeocin-induced transcriptional changes unique to *hvatr.g* plants. There were 358 such down-regulated genes among which dominated 67 histone genes of all types (2× *H1*, 17× *H2A* in different variants including two copies of *H2A.X* variant associated with marking the DNA damage sites, 17× x *H2B*, 5× *H3.1* and 26× *H4*). The down-regulated replication factors included large subunit of DNA primase, *MCM3* and GINS complex subunit *PSF1*, and cell cycle genes *MAD2*, *AURORA KINASE 1.A* (*AUR1.A*) as well as microtubule-associated protein *TORTIFOLIA1*. From the DDR genes, we found significant down-regulation of *RECQL2* helicase, RecQ-mediated genome instability protein 1 (*RMI1*), two uncharacterized DNA ligases and *F BOX-LIKE17* (*FBL17*). The FBL17 is interesting candidate as its loss of function mutant was identified in an *atr* phenotype suppressor screen in Arabidopsis and was described as *SUPPRESSORS OF ATR 1* (*SOAT*)^33^. When inspecting 164 genes up-regulated by zeocin treatment in *hvatr.g* and not in WT, we found *APURINIC ENDONUCLEASE-REDOX PROTEIN* (*ARP*) that is a major endonuclease involved in base excision repair (BER), RAD23 that has a role in NER and interacts with the 26S proteasome components, and also ESSENTIAL MEIOTIC ENDONUCLEASE 1B (EME1B) that is known to interact with endonuclease MUS81. Up-regulation of these components indicates specific shift in the use of DDR pathways towards nucleolysis, BER and NER and in *hvatr.g* plants.

## Discussion

We established in vitro DNA damage treatment conditions for barley, generated a list of barley DDR genes including the *SOG1* and *SGL* family and performed transcriptomic analysis of WT and *hvatr.g* plants in response to zeocin treatments.

A protocol for efficient induction of DNA damage under controlled in vitro conditions is not well established for barley. Our initial attempts to treat whole sterilized barley seeds according to established protocols in Arabidopsis failed. The reasons were high frequency of fungal contamination, highly variable responses to chemical treatments and complexity of root phenotype (multiple roots emerging). Based on extensive testing and modifications, we propose a protocol using dissected mature embryos and their cultivation on solid media containing genotoxins. Dissecting embryos represents an additional and demanding experimental step, but it helped in several ways. First, it greatly reduced fungal contaminations that occurred on whole seeds, possibly due to the complex surface of barley seeds. Second, it excluded endosperm which provided energy for the germinating embryo, allowing for minimal contact of the roots with the zeocin-containing media. Third, the germination showed less variation between the individuals and the experiments.

To induce DNA damage, we applied zeocin, a phleomycin D1/bleomycin-type antibiotic with radiomimetic effects. While DSBs are commonly caused by radiomimetic drugs, and bleomycin has been shown to induce DNA fragmentation^34^, research also revealed that only about 10% of DNA alterations caused by bleomycin involve both strands, and within this percentage, only a fraction are actual DSBs^35^. The ratio of SSBs to DSBs caused by bleomycin varies depending on concentration and chromatin compaction, ranging from 3:1 to 20:1^18,36,37^. Presence of both DSBs and SSBs was confirmed on DNA from bleomycin-treated barley root tissues via Comet assays^38^. Therefore, zeocin is a relatively broad-spectral DNA damage inducer that can potentially serve as a genotoxin for testing sensitivity of a wide range of DDR mutants. Induction of SSBs and to a lower extent also other non-DSB types of DNA damage by phleomycin type antibiotics^27^ also justifies use of these chemicals for treatments of *atr* mutants. Although we did not assess the amount of DNA-SBs directly in our experiments, inhibitory effects of zeocin treatments on plant growth and root tissue differentiation indicated that it was effective already at the lowest applied dose (100 µg/ml). This was further supported by up-regulation of the DDR marker genes *BRCA1* and *RAD51*. The effects on the shoot were less prominent, most likely because they were not in a direct contact with the drug-containing media.

Analysis of ATR mutant plants confirmed shorter but more seminal roots in *hvatr.g* compared to WT^21^. This is a principal difference compared to phenotypic analysis of dicots, where the primary root offers a simple proxy for the plant growth under both mock and genotoxic stress conditions. The exact reason for this phenotype is unclear but we propose several speculative models to it. Presumed replication-coupled defects in *hvatr.g* mutant plants might shift the ration between the root elongation by active cell division versus initiation of the new roots. In wild-type plants, formation of too many new roots might be suppressed via interaction of ATR with the factors that integrate DDR with cell cycle such as SOG1 and WEE1^7,39,40^. Yet another mechanism might include metabolic problems because *hvatr.g* plants exhibited a down-regulation of transmembrane transport genes, most notably the nitrate and nitrogen-compound transporters. We also observed down-regulation of 13 *EXPANSIN* genes, which could potentially impact cell growth and root number in *hvatr.g* plants. *EXPANSINS* were part of a root number quantitative trait locus in barley, although no specific gene was conclusively confirmed as the causative factor^41^. We also searched for misregulated genes known to be involved in barley root development. The only such candidate was down-regulated *PME5* (*PECTINESTERASE 11*), which has previously been linked to root length regulation^42^. However, to answer this in an unbiased way, a complex forward-directed suppressor screen would have to be carried out in the *hvatr.g* background, seeking double mutants that restore wild-type-like phenotype.

Genome-wide transcriptomics upon DNA damage proved useful in exploring plant DDR^7,22,43^. Therefore, it was interesting to observe transcriptional changes upon zeocin treatment in barley. A prominent effect was a response to oxidative stress. This is consistent with the notion that bleomycin type antibiotics, including zeocin, mediate production of superoxides and free oxygen radicals^44,45^. The upregulated enzymes included GSTUs and UGTs that use glutathione to detoxify reactive oxygen species. Glutathione is a buffer protein used against redox active molecules. In plants, some GSTUs have also strong antioxidative roles, with some classes of GSTU having peroxidase activity^46^. Furthermore, UGTs catalyze activation reactions of most secondary metabolites, including antioxidative molecules by addition of sugar moieties^47,48^.

Many zeocin-induced transcriptional changes included genes directly or indirectly associated with the cell cycle and cell division. The down-regulation of *CYCs* and *CDKs* indicated an inhibition of regular cell cycle progression. Presence of DNA damage generally leads to a halted cell cycle to gain time necessary for the repair^49^. A strong down-regulation of core histone genes and replication factors indicated reduced DNA synthesis. Many histone genes reach the peak of expression during S phase, when massive amounts of histones are needed for packaging of newly synthetized DNA. Interestingly, the down-regulation included also two DDR-associated *H2A.X* histone variant genes. Phosphorylated form of H2A.X (γH2A.X) marks the sites of active DNA damage repair^50^. Because H2A.X is present at specific genomic positions under non-DNA damaging conditions^51^, it likely that *H2A.X* is transcriptionally-regulated in the cell cycle stage-dependent manner. Our data suggest (indirectly) that the H2A.X response to DNA damage occurs at the level of post-translational modifications. An obvious consequence of the response at the protein rather than transcriptional level would be a faster reaction time. This could be important in order to pause cell cycle and start the repair before more damage occurs either directly by the mutagen or indirectly by continuation of regular cellular processes. The second prominent trend indicated a shift from mitotic cycling towards endoreduplication. Endoreduplication is a modified cell cycle where G2 phase is followed by another S-phase instead of mitosis^52^. Endoreduplication is part of a standard plant developmental program but can be alleviated by stress^39,53^. In Arabidopsis, the process is controlled by the KIP-RELATED PROTEINS (KRPs) and SIAMESE-RELATED proteins (SMRs), where the first promote mitosis while the latter endoreduplication^54^. We found zeocin-induced up-regulation of barley *SMR3*. In Arabidopsis, SMRs suppress mitosis by repressing A and B type CYCs and B type CDKs and number of these factors was downregulated by zeocin treatment in barley. The other SMR activity is to promote endoreduplication cycle by suppressing expression in the signaling cascade consisting of CYCDD, CDKA, RBR1, E2Fs and FBL17 and leading to KRPs^54^. In barley, we found transcriptionally down-regulated ERF factor *DEL1* and *FBL17*. Barley plants show generally low levels of endoreduplication in somatic tissues under normal conditions but the frequency is higher in specialized tissues such as some endosperm developmental stages^20^. Interestingly, there was a significant increase in the frequency of endoreduplicated nuclei in the roots of zeocin-treated plants. This is in agreement with the molecular signatures in our RNA-seq analysis and suggests that barley performs adjustments to its cell cycle in responses to genotoxic stress via evolutionarily conserved SMR-dependent pathway.

To focus RNA-seq on DDR components, we created list of barley DDR genes using protein homologies with Arabidopsis candidates^22^. Through selection, filtering and manual curation steps, we identified 421 barley candidates. Some, like the barley *BRCA1* homolog had lower similarity to Arabidopsis and were added manually. The list also included homologs of Arabidopsis DDR response master regulator *SOG1*, and its closely related genes *ANAC044* and *ANAC085*^26,55^. We found five members of this family in barley. Comparisons with rice and maize suggested that SOG1 is a single copy gene in barley. In contrast, the other four family members clustered with one clade with the Arabidopsis ANAC044, ANAC085 and rice SGL. This suggested that these copies represent barley *SGLs*. Interestingly, *SGLs* were more transcriptionally responsive to zeocin-induced DNA damage than SOG1 in barley. This aligns with Arabidopsis, where SOG1 shows minimal transcriptional changes to DNA damage, but ANACs are up-regulated^7^, and indicates an evolutionarily conserved regulation of SOG1 at the protein level and *SGLs* at the transcriptional level during DDR. Other up-regulated genes included several DNA damage repair factors and positive regulators of HR including *RAD51*, *BRCA1*, *RecQL3*, *NSE4.A*, *GMI1*, or *BCP1*.

Transcriptomic data of *hvatr.g* showed activated DDR even under the mock conditions, consistent with previous findings of increased DNA damage in non-treated *hvatr.g* plants^21^. Up-regulated factors indicated greater utilization of mismatch and NER pathways in ATR mutants. Notably, moderately up-regulated genes in *hvatr.g* included *RAD23* and *CDC48*. RAD23 is connected to proteolysis helping with the cell-cycle progression and stress response^56^. In yeast, Rad23 recognizes a complex of Cdc48 and Ubiquitin ligase E4, facilitating proteasomal degradation. Plant CDC48 homologs have similar roles in protein degradation^57^. The enrichment of genes involved in ubiquitinilation, along with down-regulation of E2 Ub-conjugating enzymes and RGLG2 Ub-ligase E3 in *hvatr.g*, suggests increased protein turnover. Alternatively, elevated CDC48 expression may contribute to methionine metabolism for cell defense in the nicotineamine pathway or facilitate de-condensation of centromeric heterochromatin and/or activation of rDNA genes as observed in Arabidopsis. These pathways potentially have support from other upregulated genes. Notably, *FBL17* was down-regulated in *hvatr.g*, and Arabidopsis *FBL17* mutants were identified as *SUPPRESSORS OF ATR 1* sensitivity phenotype^33^, indicating a transcriptional regulatory feedback loop between ATR and FBL17.

Besides of the obvious transcriptional differences between *hvatr.g* and wild-type, it has to be noted that vast majority of the transcriptional response between both genotypes remained unchanged. This is most likely due to a partial functional redundancy of plant ATM and ATR kinases as described in Arabidopsis^58^. Therefore, a barley *atm atr* double mutant would be needed to uncover the whole spectrum of genes controlled by these key DDR kinases. Alternatively, analysis of barley *sog1* mutant might be methodologically easier and possibly even more informative option^7,8^

In conclusion, our study indicates that barley exhibits a conserved response to chemically-induced DNA-SBs. We observed molecular signatures of oxidative stress response, that is consistent with the zeocin expected mode of action and responses to both DNA single and double strand breaks. We also identifies some genes that could be possible targets of modifying mitotic division and endoreduplication in barley. This study offers valuable resources for further detailed investigations into barley's DDR, its associations with other stresses, and plant development.

## Materials and methods

### Plant materials and growth conditions

We used barley cultivars Golden Promise and Sebastian (WT), and *hvatr.g* TILLING mutant allele of *ATR* (HORVU.MOREX.r3.7HG0748510) gene^21^. In vitro plant cultivation was done in an air-conditioned phytochamber with a long day regime (16 h light, 150 µmol m^−2^ s^−1^, 21 °C, 8 h dark, 19 °C). Plants used for seed production were grown in the climate-controlled phytotron under long day conditions (16 h light, 150 μmol m^−2^ s^−1^, 15 °C, 8 h dark, 13 °C, 65% humidity). Plant materials used in this work are a part of cultivated cereal varieties and were not resourced in field. Golden Promise (Acc. No. HOR 16645) is available at the Leibniz Institute of Plant Genetics and Crop Plant Research, IPK, Genebank, Gatersleben, Germany and Sebastian (Acc. No. 03C0602773) at the Germplasm Resource Information Network (GRIN), Prague, Czech Republic. Mutant *hvatr.g* is available at *Hor*TILLUS (*Hordeum*—TILLING—University of Silesia) database upon request from M.S. Experimental research on plant material in this study, including its collection, complied with the relevant institutional, national, and international guidelines and legislation.

### DNA damage assays

Barley seeds were surface sterilized with 70% ethanol (v/v) for 5 min, followed by 10 min treatment with 8% sodium hypochlorite (v/v) and final triple wash with ddH_2_O. Sterilized seeds were imbibed overnight in sterile water at 4 °C in dark. The following day, embryos were carefully excised under binocular in a sterile laminal flow-hood from the seeds and placed scutellum side down on ½ MS medium with 0.6% agarose (w/v) or medium supplemented with zeocin (ThermoFisher Scientific, Cat. no. R25001) in 107 × 94 × 96 mm boxes (Duchefa, Cat. no. S1686). In place of a lid, another container was sealed to the one containing medium with embryos with a parafilm (total height 192 mm). Containers were placed in a phytochamber (Percival Scientific) and plants were grown for 14 days after which they were carefully pulled out of the medium for measurements. All the measurements were completed using the ImageJ plugin SmartRoot^59^. Experiments were performed in three biological replicates. For testing the different concentrations of zeocin required for wild-type barley (*cv.* Golden Promise) phenotypic response each replicate contained 18–20 individual plants. In the DNA damage assays comparing the phenotypic response to zeocein of *hvatr.g* and wild-type *cv.* Sebastian, each replicate had 9–10 plants per genotype. Statistical significance was tested with One-way ANOVA with post hoc Tukey HSD in Minitab (www.minitab.com).

### Propidium iodide staining and root microscopy

Barley plants *cv.* Golden Promise grown for seven days on a control or 100 μg/ml zeocin media were used to assess root morphology at microscopic level. Three representative plant samples were chosen for imaging. The whole root was stained by pseudo-Schiff propidium iodide staining as described (Coiro and Truernit^60^). Incubation with propidium iodide and Schiff reagent lasted for 48 h. Following the overnight incubation with chloral hydrate solution roots were mounted on glass in water. Imaging was performed using Leica TCS SP8 STED3X confocal microscope (Leica Microsystems, Wetzlar, Germany), equipped with an HC PL APO CS2 10×/0,4 DRY objective, hybrid detectors (HyD), and the Leica Application Suite X (LAS-X) software version 3.5.5 with the Leica Lightning module (Leica, Buffalo Grove, IL, USA). For propidium iodide acquisition, laser excitation at 534 nm and emission at 550–730 nm were used. The maximal projection pictures were constructed from aligned Z-stack images of approximately 250–300 μm steps, containing 45 individual optical sections. The images were post-processed by Leica Lightening software module.

### Flow-cytometry

The nuclear ploidy measurements were done on 20 Golden Promise plants grown from dissected embryos for seven days on solid ½ MS medium with or without 300 μg/ml zeocin. Two to three root apical meristems from each individual plant were chopped using a razor blade directly into 500 ml Otto I buffer (0.1 M citric acid, 0.5% Tween 20 v/v). Nuclei suspension was filtered through a 50 µm nylon mesh into a fresh tube, mixed with 1 ml of Otto II buffer (0.4 M Na_2_HPO_4_ × 12H_2_O) containing 2 μg DAPI (4′,6-diamidino-2-phenylindole) fluorescent stain. Ploidy was measured on a Partec PAS I flow cytometer with WT barley leaf tissue as a standard. Statistical significance was assessed by a two-sample T-Test in Minitab.

### Reverse transcription-quantitative polymerase chain reaction (RT-qPCR)

Golden Promise seeds were surface sterilized, embryos dissected, and grown on ½ MS medium with 0.6% agarose. After 7 days, young seedlings were moved to liquid ½ MS medium without or with zeocin (300 μg/ml). Sampling was performed at strict time points 0.5, 1 and 6 h after the beginning of treatment. All root apical meristems (RAMs) from a single plant were dissected and flash-frozen in liquid nitrogen. Three plants were taken for each treatment and stored until use at − 80 °C. RNA extraction was performed by RNeasy Mini Kit (Qiagen, Cat. no. 74104) according to manufacturer’s instructions with on column DNAse I treatment. cDNA was constructed with RevertAid H Minus First Strand cDNA Synthesis Kit (Thermo Scientific™, Cat. no. K1631). The qPCR was performed with the HOT FIREPol® EvaGreen® qPCR Mix Plus (Solis BioDyne, Cat. no. 08-24-205 0000S) in CFX96 Touch Real-Time PCR Detection System (BioRad). Mann–Whitney U-test was performed in Minitab to assess statistical significance of the data.

### RNA-sequencing

Plant material for RNA-sequencing was prepared similarly to RT-qPCR experiments with following modifications: 500 μg/ml zeocin treatment was applied for 6 h. Genotypes used were wild-type *cv.* Sebastian and mutant *hvatr.g*. Quality of the total RNA was checked on BioAnalyzer 2100 with RNA 6000 Pico Chips (Agilent) and samples with RNA integrity number > 8.1 were processed further. RNA sequencing was performed in three biological replicates for every experimental point at Novogene (UK) Company Limited using 150 bp paired-end protocol. At least 60 million paired-end reads were produced for each sample. The sequencing reads were deposited at Gene Expression Omnibus under ID GSE235051.The raw reads were trimmed using Trim Galore v.0.4.1 (www.bioinformatics.babraham.ac.uk/projects/trim_galore) and aligned to the 3rd version of reference genome of the *H. vulgare* cv. Morex^61^ using HiSat2 v.2.1.0 genomic mapper^62^. Aligned reads were assigned to the genes according to the genome annotation using Subread v.1.5.2 software^63^ and raw read counts were normalized to TPM expression levels. Differential expression analysis was performed using DESeq2 v.1.38.3 package^64^ in R v.4.2.2 software (www.r-project.org). DEGs were identified according to the Benjamini–Hochberg FDR-adjusted *p*-value (< 0.05).

### GO Term enrichment analysis

For the assessment of Gene Ontology an online tool gProfiler was used (www.biit.cs.ut.ee/gprofiler/gost). The tool uses g:SCS algorithm for correction of *p*-values. Output was manually curated to filter out redundant GO terms.

### Identification of DNA damage response and repair genes in barley

The amino acid sequences of 321 Arabidopsis DNA damage repair genes^22^ were BLASTed^65^ to the set of 83,661 barley genes^66^. Subsequently, all barley candidates were BLASTed back to Arabidopsis to confirm best similarity. The candidates confirmed in both directions of reciprocal BLAST were taken for further analysis. They were filtered by the BLAST E-value (≤ 0.01), comparison of protein lengths, and alignment lengths (40% and more was accepted for both parameters).

### Phylogeny

The amino acid sequences of NAC domains found in *A. thaliana* SOG1, ANAC044, ANAC85, ANAC005, ANAC006; *Oryza sativa* SOG1, SGL, *Zea mays* NACTF99, NACTF08, and *Hordeum vulgare* HORVU.MOREX.r3.2HG0175540 (*SGL.B*), HORVU.MOREX.r3.2HG0175550 (*SGL.C*), HORVU.MOREX.r3.2HG0175560 (*SGL.D*), HORVU.MOREX.r3.6HG0590960 (*SGL.A*), HORVU.MOREX.r3.7HG0670800 (*SOG1*) were retrieved from The Arabidopsis Information Resource (TAIR, www.arabidopsis.org), The Rice Annotation Project Database (RAP-DP, www.rapdb.dna.affrc.go.jp), Maize Genetics and Genomics Database (www.maizegdb.org) and BARLEX (www.barlex.barleysequence.org) databases respectively, all accessed on May 2nd, 2023. The alignments of proteins were performed in MEGA software (www.megasoftware.net) using MUSCLE. Prepared alignments were graphically shown using SnapGene (www.snapgene.com), amino acids were highlighted based on consensus sequence and their physicochemical properties. The maximum likelihood phylogeny reconstruction was computed with MEGA using bootstrap method with 1000 iterations. The substitution model used was JTT with gamma distributed rates with five categories (+G).

### Supplementary Information

Supplementary Information 1.

Supplementary Information 2.

Supplementary Information 3.

Supplementary Information 4.

Supplementary Information 5.

Supplementary Figures.

## Data Availability

The datasets presented in this manuscript can be found in the text and figures, supplementary materials and RNA-seq reads were deposited in the NCBI Gene Expression Omnibus online repository under the number: GSE235051. The following barley genes and/or their products were mentioned in this study: *ASF1B* (HORVU.MOREX.r3.1HG0084850), *ATR* (HORVU.MOREX.r3.7HG0748510), *BARD1* (HORVU.MOREX.r3.2HG0181390), *BCP1* (HORVU.MOREX.r3.6HG0554520), BRCA1 (HORVU.MOREX.r3.1HG0078370), CAX5 (HORVU.MOREX.r3.4HG0337640.1), CDC48 (HORVU.MOREX.r3.2HG0105790, HORVU.MOREX.r3. 2HG0105790), CDKB1;2 (HORVU.MOREX.r3.5HG0463930), CDKB2;2 (HORVU.MOREX.r3.4HG0384440), CHR1.B (HORVU.MOREX.r3.4HG0338270), CMT3 (HORVU.MOREX.r3.6HG0628050), CYC6B (HORVU.MOREX.r3.3HG0301080), CYCA1.A (HORVU.MOREX.r3.3HG0249410), CYCA3;1.A (HORVU.MOREX.r3.4HG0342640), CYCB1;2.A (HORVU.MOREX.r3.3HG0259030), CYCB1;2.B (HORVU.MOREX.r3.1HG0069480), CYCB1;2.C (HORVU.MOREX.r3.3HG0295540), CYCB1;3.A (HORVU.MOREX.r3.1HG0069490), CYCB1;3.A (HORVU.MOREX.r3.1HG0069550), CYCB2;3.B (HORVU.MOREX.r3.7HG0751620), CYCD3;3 (HORVU.MOREX.r3.5HG0467900), FAS1 (HORVU.MOREX.r3.5HG0501270), FBL17.A/SOAT1 (HORVU.MOREX.r3.5HG0433490), GMI1 (HORVU.MOREX.r3.1HG0058570), MET1 (HORVU.MOREX.r3.2HG0151710), MRE11 (HORVU.MOREX.r3.7HG0715110), MSH5 (HORVU.MOREX.r3.1HG0068200), NICOTIANAMINE SYNTHASE (HORVU.MOREX.r3.4HG0415050), NSE4.A (HORVU.MOREX.r3.7HG0724600), PME5 (HORVU.MOREX.r3.2HG0189320.1), RAD23 (HORVU.MOREX.r3.6HG0569510), RAD51 (HORVU.MOREX.r3.7HG0721560), RecQL 3 (HORVU.MOREX.r3.6HG0620770), RGLG2 (HORVU.MOREX.r3.7HG0677600), RPA1A (HORVU.MOREX.r3.6HG0620000), RPA2.K (HORVU.MOREX.r3.6HG0597130), RPA70C.C (HORVU.MOREX.r3.1HG0021720), SGL.A (HORVU.MOREX.r3.6HG0590960), SGL.D (HORVU.MOREX.r3.2HG0175560), SGL.C (HORVU.MOREX.r3.2HG0175550), SGL.B (HORVU.MOREX.r3.2HG0175540), SOG1 (HORVU.MOREX.r3.7HG0670800), TFIIB (HORVU.MOREX.r3.1HG0079200), TFIIIA (HORVU.MOREX.r3.6HG0548800), UBC2 (HORVU.MOREX.r3.5HG0517500), Ub-LIGASE E3 (HORVU.MOREX.r3.3HG0229520), VIM1 (HORVU.MOREX.r3.1HG0000630). Additionally, the following non-barley genes and/or their products were mentioned in this work. The Arabidopsis ANAC044 (AT3G01600), ANAC085 (At5g14490), SOG1 (AT1G25580). The rice (*Oryza sativa*) SOG1 (Os06g0267500) and SGL (Os02g0594800) and the maize (*Zea mays*) NACTF99 (Zm00001eb280480) and NACTF8 (Zm00001eb280490).
