# Supplementary material for: Zeocin-induced DNA damage response in barley and its dependence on ATR
Source: Sci Rep. 2024 Feb 7;14:3119. doi: 10.1038/s41598-024-53264-0 (PMC10850495; doi:10.1038/s41598-024-53264-0)
Supplement: Supplementary file 6 — Supplementary Figures. [file 41598_2024_53264_MOESM6_ESM.docx]

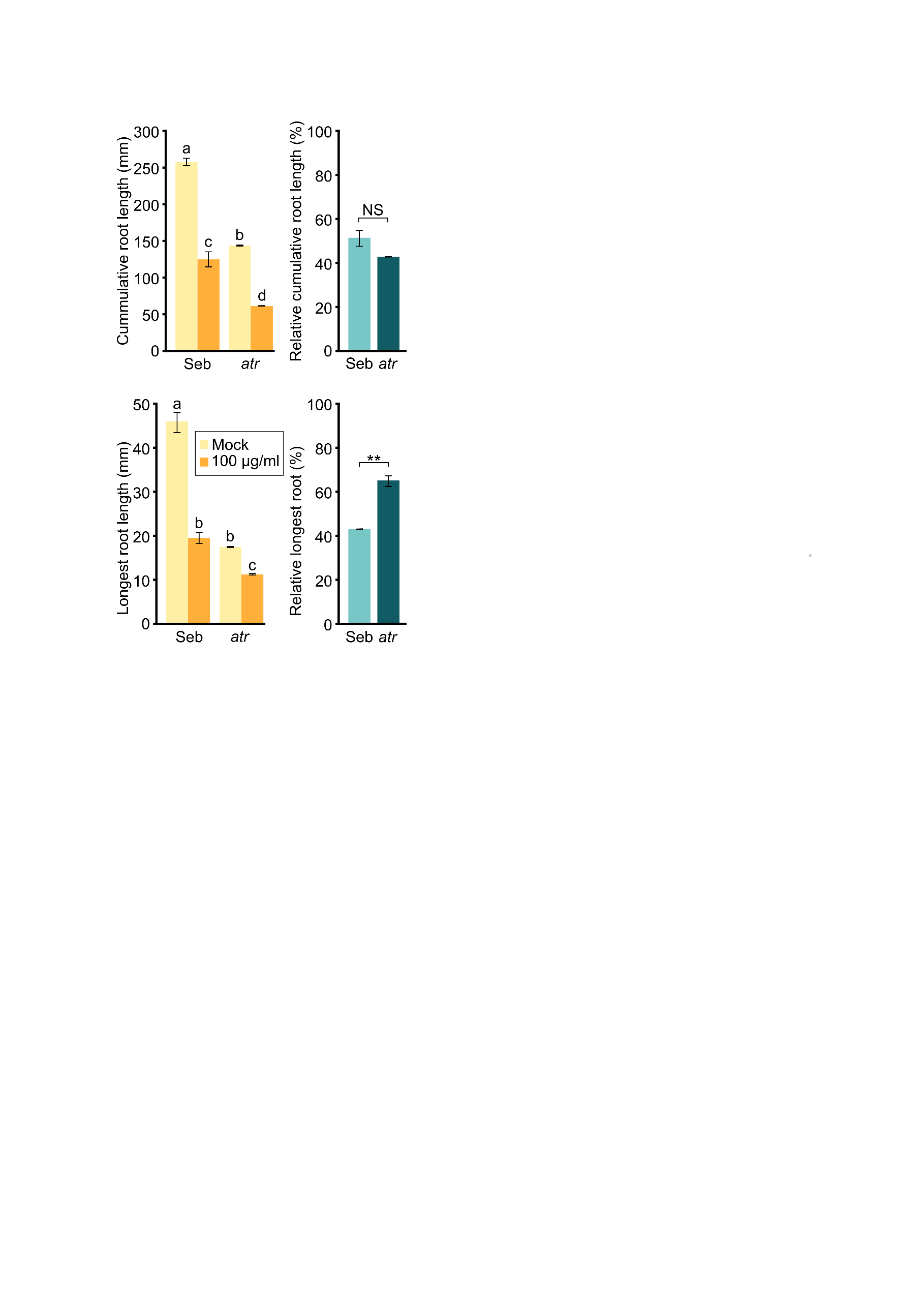


**Supplemental Figure 1**: Measurements of additional phenotypic traits showing the effect of zeocin treatment on Seb and *atr* plants’ growth. Error bars indicate the standard deviation between the means of three biological replicates. For the absolute values represented the letters above columns indicate similarities between samples. The same letters indicate samples that were not significantly different in one-way ANOVA with *post-hoc* Tukey’s test (*P* < 0.05). For the assessment of the statistical significance of the relative values the to-sample T-test was used, * *P* < 0.05, ** *P* < 0.01

**Supplemental Figure 1**


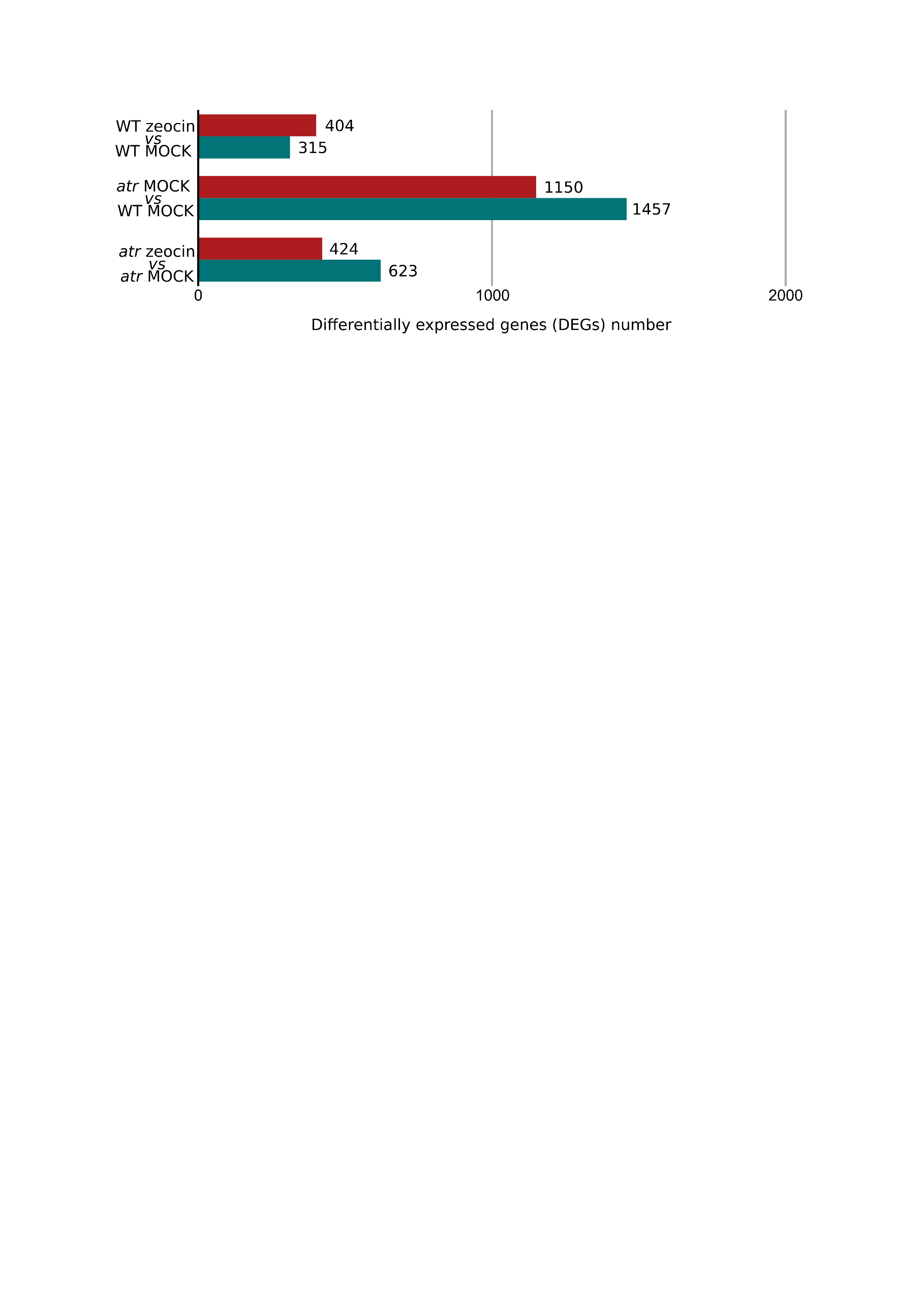


**Supplemental Figure 2**: Bar plot showing the number of differentially expressed genes (DEGs) found in the transcriptome comparison of wild-type Sebastian treated and untreated plants (WT MOCK vs WT zeocin), *atr* mutant and wild-type plants (*atr* MOCK vs WT MOCK) and *atr* mutant treated and untreated plants (*atr* MOCK vs *atr* zeocin). Up-regulated genes are presented by the red bar, and down regulated by the blue bar.

**Supplemental Figure 2**


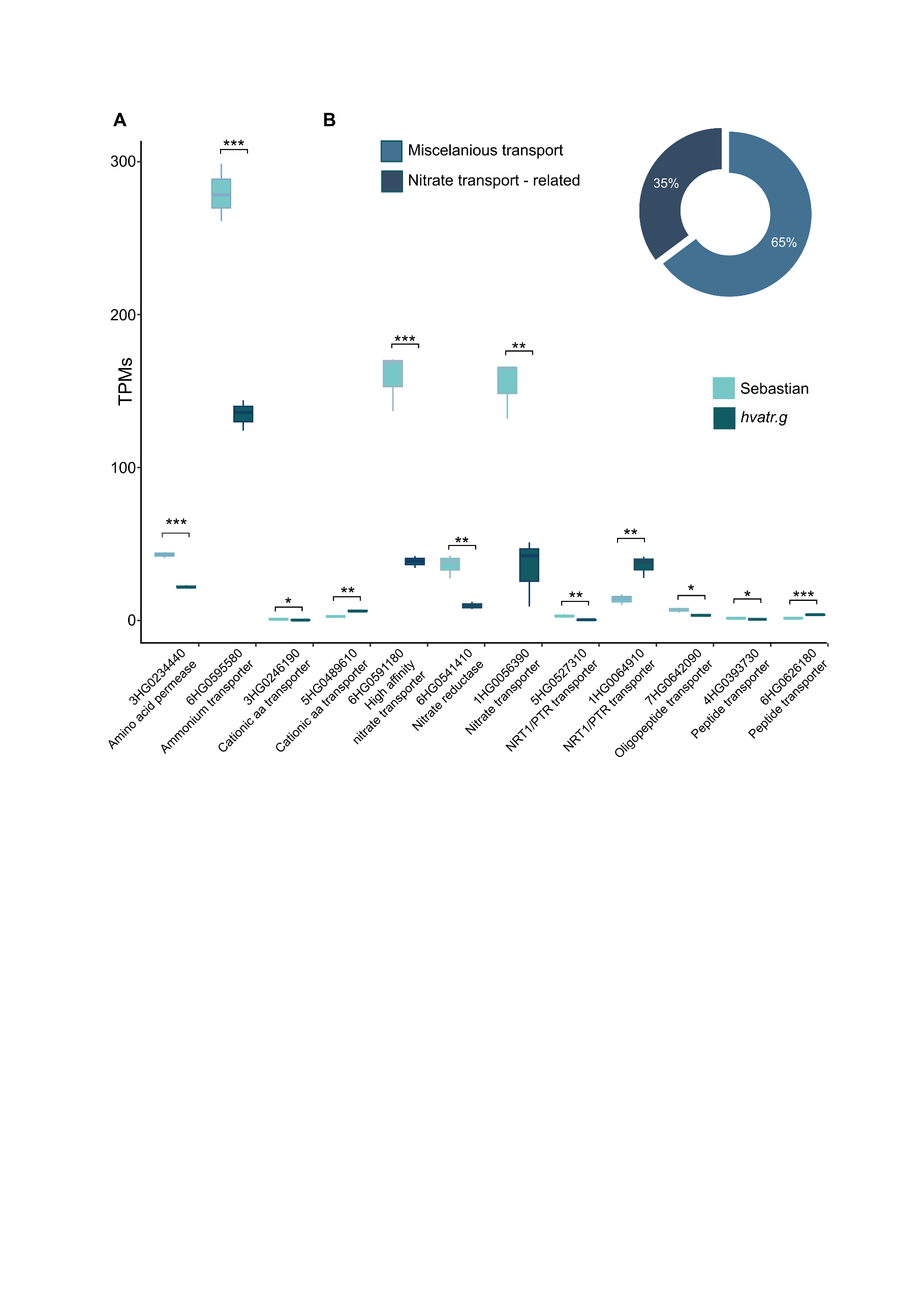


**Supplemental Figure 3**

**Supplemental Figure 3**: (**A**) Bar and whiskers plot showing the expression of selected genes related to nitrate transport in Sebastian (wild type) and *hvatr.g* in transcripts per million (TPMs). The boxplots’ hinges are in the 1st and 3rd quartile, with a marked median. Whisker marks show the lowest or highest value within the 1.5 interquartile range below or above hinges. Asterisks represent significant differences in T-test, *** *P < 0.05,* *** P* < 0.01, **** P* < 0.05. (**B**) Pie chart showing the percentage of down-regulated genes involved in Transmembrane transport (GO:0055085) involved in nitrate transport.

**Supplemental Figure 3**


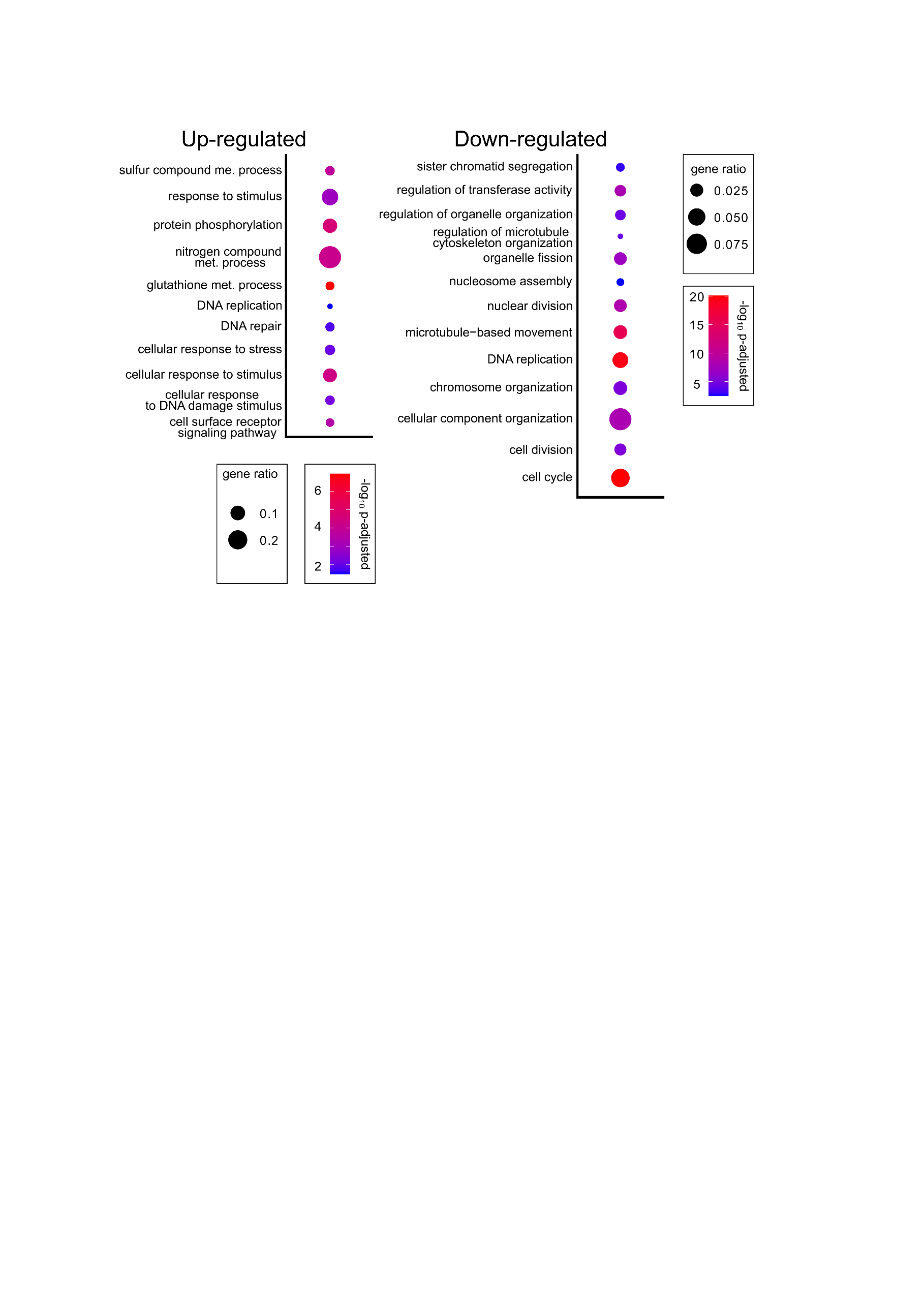


**Supplemental Figure 4**

**Supplemental figure 4:** Biological processes enriched among significantly up-regulated and down-regulated genes in zeocin versus mock treated *hvatr.g* plants. Redundant GO terms were removed manually, based on P – value. The full list of GO terms can be found in Suppl. Dataset 3. Statistical significance was determined by Fisher’s one-tailed test with g:SCS algorithm correction. Gene ratio represents the number of genes found in the category compared to the total number of genes in the query.

**Supplemental Figure 3**: Gene enrichment analysis for biological processes in *atr* mutant treated with zeocin compared to the untreated mutant plants. Redundant GO terms were removed manually, based on *P* – value. The full list of GO terms can be found in Suppl. Dataset 3. Statistical significance was determined by Fisher’s one-tailed test with g:SCS algorithm correction. Gene ratio represents the number of genes found in the category compared to the total number of genes in the query.
